# The interplay between the anticipation and subsequent online processing of emotional stimuli as measured by pupillary dilatation: the role of cognitive reappraisal

**DOI:** 10.3389/fpsyg.2014.00207

**Published:** 2014-03-13

**Authors:** Marie-Anne Vanderhasselt, Jonathan Remue, Kwun Kei Ng, Rudi De Raedt

**Affiliations:** ^1^Psychopathology and Affective Neuroscience Laboratory, Department of Experimental Clinical and Health Psychology, Ghent UniversityGhent, Belgium; ^2^Brain and Behaviour Lab, Department of Psychology, National University of SingaporeSingapore, Singapore

**Keywords:** pupil diameter, emotion anticipation, online emotional processing, cognitive reappraisal

## Abstract

Emotions can occur during an emotion-eliciting event, but they can also arise when anticipating the event. We used pupillary responses, as a measure of effortful cognitive processing, to test whether the anticipation of an emotional stimulus (positive and negative) influences the subsequent online processing of that emotional stimulus. Moreover, we tested whether individual differences in the habitual use of emotion regulation strategies are associated with pupillary responses during the anticipation and/or online processing of this emotional stimulus. Our results show that, both for positive and negative stimuli, pupillary diameter during the anticipation of emotion-eliciting events is inversely and strongly correlated to pupillary responses during the emotional image presentation. The variance in this temporal interplay between anticipation and online processing was related to individual differences in emotion regulation. Specifically, the results show that high reappraisal scores are related to larger pupil diameter during the anticipation which is related to smaller pupillary responses during the online processing of emotion-eliciting events. The habitual use of expressive suppression was not associated to pupillary responses in the anticipation and subsequent online processing of emotional stimuli. Taken together, the current data suggest (most strongly for individuals scoring high on the habitual use of reappraisal) that larger pupillary responses during the anticipation of an emotional stimulus are indicative of a sustained attentional set activation to prepare for an upcoming emotional stimulus, which subsequently directs a reduced need to cognitively process that emotional event. Hence, because the habitual use of reappraisal is known to have a positive influence on emotional well-being, the interplay between anticipation and online processing of emotional stimuli might be a significant marker of this well-being.

## Introduction

More than a century ago, emotions have been defined as people's valenced (positive and negative) reactions to events that they perceive as relevant to their ongoing concerns (James, 1884). Emotions are complex psychological states that involve cognitions and embodied (physiological) sensations, the latter referring to relevant sensory-motor and somatic states that are necessary for encoding and interpretation (Niedenthal et al., 2005a). Even though emotions can sometimes have an overwhelming influence on thought and behavior, people have—to some extent—the ability to influence and control which emotion they have, when and where they have them (Gross, 1998). A vibrant research field has emerged, investigating the nature and effects of different forms of emotion regulation involved in the occurrence, intensity, and duration of emotional states (for reviews, see Gross and Thompson, 2007; Koole, 2009), with cognitive reappraisal and expressive suppression being the most studied strategies that are using regulatory (cognitive) effort. During *cognitive reappraisal*, people attempt to rethink their cognitive appraisals to reduce the emotional impact of the emotion-eliciting event (Gross, 1998, 2002). By using *expressive suppression*, on the other hand, people aim to achieve response modulation whereby an individual voluntarily inhibits emotional expressive behavior (e.g., motor or bodily responses to the emotion-eliciting event). Importantly, the habitual use of these emotion regulation strategies seems to have a crucial influence on emotional states: the habitual use of cognitive reappraisal is assumed to be an important factor in determining higher well-being (Thompson, 1991; Cicchetti et al., 1995), whereas habitual suppressors generally report greater negative affect (Gross and John, 2003). Moreover, these emotion regulation strategies play a crucial role in psychopathology and its treatment (Gross and John, 2003).

Interestingly, emotions can occur during an emotion-eliciting event, for example a presentation on a scientific meeting, but they can also arise when anticipating an emotion-eliciting event, for example while thinking of a pending presentation. Indeed, the anticipation and representation of the meaning of an upcoming emotional event can already lead to the experience of emotions, which trigger emotion systems (e.g., embodiment) similar to those systems that become activated during the online emotion processing (“offline” embodiment of emotion, Niedenthal et al., 2005b). Moreover, future expectations elicit similar emotional and physiological stress responses as stressful events themselves (Gramer and Reitbauer, 2010; Waugh et al., 2010). Although this anticipatory aspect of emotion is largely underexplored, individuals might benefit from this emotion anticipation to flexibly adapt to changing cognitive situations and social demands. Moreover, as stated by the Dual Mechanisms of Control (DMC) theoretical framework, the ability to proactively prepare for upcoming contexts contributes to the successful completion of tasks that require extra effortful (cognitive) control or regulation (Braver et al., 2007; Braver, 2012). In other words, the cognitive effort that is generated during the anticipation of specific events seems to influence the cognitive effort that is necessary to process that emotion-eliciting event later on. Even though this latter theoretical framework is discussed in the context of conflict processing (and not emotional information), one can argue that emotional stimuli interfere with ongoing cognitive processing and require additional cognitive processing effort to cope with the emotional content (Etkin et al., 2006; Cohen et al., 2011). Therefore, it is important to investigate whether the way individuals anticipate an emotional stimulus influences the subsequent online processing of that event.

Pupil diameter is known to be a physiological indicator of activity in the sympathetic nervous system. Pupil constriction (a decrease in pupil diameter relative to baseline) is mostly driven by the parasympathetic division of the autonomic nervous system, and pupil dilation is mostly driven by the sympathetic division (Loewenfeld, 1993). Bradley et al. (2008) demonstrated that emotional arousal enlarged the pupil size, independent of the valence of the emotion. Moreover, in line with the putative arousing nature of cognitive control, an increased pupil dilatation has been shown to be a reliable measure of the extent of mental effort (Hess and Polt, 1964; Moresi et al., 2008), with a positive association between cognitive load and pupil dilatation (Steenhauer et al., 2000; see Kahneman, 1973). Indeed, pupil dilatation seems to reflect activity in the locus coeruleus-norepinephrine system (Jepma and Nieuwenhuis, 2011; Murphy et al., 2011), an arousal-related neurochemical system that is thought to play a key role in the cognitive control of behavior (Aston-Jones and Cohen, 2005). Based on the link between arousal and mental effort (van Steenbergen and Band, 2013), pupillary activity has been proposed as a physiological marker of emotion regulation (e.g., Siegle et al., 2003; Urry et al., 2006; van Reekum et al., 2007). In several studies, larger pupil dilation has been related to more cognitive top-down effort exerted to cognitively process and regulate the emotional stimulus (Kahneman and Beatty, 1966; Siegle et al., 2003; Ohira et al., 2006; Allard et al., 2010). Johnstone et al. (2007), for example, demonstrated that—in healthy controls—emotion regulation effort was associated with greater pupil dilatation, and with decreased activation in limbic areas. All together, pupil size enlarges in response to automatic responses (i.e., arousal) to valenced stimuli, but is furthermore related to the extent of mental effort (or the level of central nervous system processing allocated to a task) to control over automatic responses. Interestingly, pupil size is also a reliable measure of cognitive processing effort during the anticipation of events. Research has shown that anticipatory cognitive processing effort is associated with increased pupil size (Moresi et al., 2008), both for aversive and neutral events (Bitsios et al., 2004). The more processing load or mental effort that is needed for the upcoming task, the larger are the pupillary dilations during the anticipation phase (Moresi et al., 2008). Pupil size (as an objective index of arousal associated with mental efforts) seems thus a suitable technique to investigate cognitive efforts that individuals invest during the anticipation and the online processing period of emotional stimuli.

Hence, in this study, a sample of healthy volunteers was submitted to an appraisal paradigm during which we asked them to respond naturally to emotion-eliciting images (participants were not aware of the aim of the study). Before the presentation of each image, we informed them about the valence of the upcoming image so that they could anticipate their emotional reaction. Processing of emotional stimuli requires cognitive resources (Pessoa et al., 2002; Okon-Singer et al., 2007), and we measured the physiological response of the pupil during both the instructional cue and the emotional picture during an appraisal paradigm. The overall purpose of this study is twofold: *First*, we tested (using pupillary responses as an index of cognitive processing effort) whether the anticipation of an explicit positive and negative emotional stimulus would influence the subsequent online processing of that emotional stimulus. We expected, based on the DMC theoretical framework (Braver et al., 2007), a negative correlation between pupil size during the anticipation and during the online processing of the emotion-eliciting event. *Second*, we tested whether individual differences in the habitual use of emotion regulation strategies, measured using the Emotion Regulation Questionnaire (ERQ, Gross and John, 2003), are associated to pupillary responses during the anticipation and online processing of an emotional stimulus. Cognitive reappraisal is an antecedent-focused strategy which starts operating early in the emotion generation process (Sheppes and Gross, 2011), and this processing gradually diminishes over time when the emotional stimulus is still presented (Goldin et al., 2008). Therefore, we expected habitual reappraisers to demonstrate larger pupillary responses when anticipating an emotional stimulus, which might be inversely related to the cognitive effort during the online emotion processing phase. Although reappraisal has been mainly related to coping with negative content, it has been argued that people attempt to regulate all their emotional responses—also positive ones—to regain an emotional balance, and to avoid mood incongruence (Parrot and Sabini, 1990). For habitual expressive suppressors, on the other hand, we did not expect an effect on the anticipation of an emotional stimulus, but we expected larger pupillary responses during the online processing of the emotional stimulus. This is because expressive suppression is a response-focused strategy which starts operating when emotional response tendencies have been fully activated, and therefore solely increase processing effort when confronted with the emotion-eliciting event. Because healthy volunteers regulate their emotions in order to maintain an emotional balance (Parrot and Sabini, 1990), we had no explicit hypothesis regarding the emotional specificity of the pupillary responses during the anticipation and online processing of positive and negative material.

## Materials and methods

### Participants

A sample of 55 college undergraduates of Ghent University participated as part of a course requirement. Ages ranged from 17 to 36 years (*M* = 19.71, *SD* = 3.76). All participants (8 males and 47 females) were right-handed, and none reported a history of or currently had a neurological or psychiatric illness. Moreover, participants were excluded if they reported a history of serious head injury, or were having eye problems or difficulties in vision not corrected by the use of glasses or contact lenses. Participants gave their written informed consent and received credits for their participation. The study was approved by the ethics committee of the Ghent University (2012/36-10/7/12). This study was part of a larger project investigating other neuro-cognitive markers of emotion regulation and self-esteem.

### Material

#### Emotion-regulation questionnaire (ERQ, Gross and John, 2003)

The ERQ was administered to measure the habitual use of two different emotion regulation strategies: cognitive reappraisal and expressive suppression. The ERQ consists of 10 items rated on a scale from 1 (strongly disagree) to 7 (strongly agree). Reappraisal scores are calculated by six items of the ERQ, and suppression scores are calculated by the remaining four items of the ERQ. Evidence has shown good predictive ecological validity for emotion regulation, such as the ability to down-regulate anger (Mauss et al., 2007).

#### Appraisal paradigm

Stimulus presentation was programmed in Tobii software. All stimuli were presented on a computer screen and participants were seated at a distance of 60 cm from the screen. Participants were presented with a set of 30 pleasant (typically depicting friendship and love) and 30 unpleasant (typically depicting violence and physical/emotional pain) gray scaled photographs from the International Affective Picture System (IAPS; Lang et al., 2007)^1^. Pictures were selected on the basis of IAPS normative data, across men and women. According to the IAPS norms, pleasant and unpleasant pictures differed on valence ratings (on a 1–9 scale where 9 signifies completely happy: positive = 7.35 ± 0.57; negative = 2.52 ± 70), *t*_(58)_ = 29.25, *p* < 0.001, and arousal ratings (on a 1–9 scale where 9 signifies completely aroused: positive = 4.70 ± 0.59; negative = 6.16±.52), *t*_(58)_ = 10.41, *p* < 0.001. These gray-scaled images were corrected to have similar brightness or luminance values (based on the log-averaged pixel values): *M* negative = 90.35, ±30.88; *M* positive = 104.38, ±23.61, *p*s > 0.1. The luminance value of the cue negative (125.85) and cue positive (125.99) were very similar.

Picture presentation (both negative and positive) order was pseudo randomized with the constraint that no more than three pictures of the same valence were shown consecutively. Before each positive or negative picture (presented for 6 s), a cue (presented for 6 s) informed participants whether the upcoming stimulus would elicit positive or negative emotions. This cue information (i.e., the word “positive” or the word “negative”) was always correct. Pictures were arranged in blocks of 20 trials in each block (three blocks in total), with 10 pictures of each hedonic content (pleasant and unpleasant) preceded by an informative cue.

Each participant was instructed that a series of pictures would be displayed and that each picture should be viewed the entire time it was on the screen. They were instructed to respond naturally to the emotional picture. They were also told that the cue before the picture presentation is to inform them about the pleasantness of the upcoming image. Based on this passive viewing paradigm, we measured pupil responses following the cue and following the target, for both emotional valences, and how cue/picture pupillary responses would interact with each other.

#### Mood

In order to evaluate temporary changes in mood before (Tpre), vs. immediately after (Tpost) the appraisal paradigm, mood ratings were administered using six visual analogue scales (VAS) providing measures of fatigue, tension, anger, vigor, depression and cheerful (McCormack et al., 1988). Participants were asked to describe how they felt “at that moment” by indicating on horizontal 100 cm lines whether they experienced the five above-mentioned mood states, from “totally not” to “very much.”

### Pupil data acquisition

A video camera and infrared light source (Tobii-TX300 eye tracking system) were directed at the participant's eye in order to track the size of the pupil. Diameter of the pupil was monitored at 300 Hz (every 3.3 ms) during the entire experiment, which resulted in approximately 1800 timepoints for each participant per condition (i.e., cue-positive; cue-negative; picture-positive; picture-negative), each baseline-corrected and averaged separately. These data passed digitally from the eye-tracker to a computer to store the acquired data along with signals marking the beginning and end of each trial.

To calibrate the eye tracker at the beginning of the pupil assessment session participants were asked to focus their attention on each of nine dots presented in a random order in either one of the four corners of the display space, midway between each corner, and in the middle of the screen.

### Pupil preprocessing

Individual data were first scanned for overall quality. Data preprocessing and analysis was performed on data averaged across both eyes (both eyes are highly correlates, *p*s > 0.8). All participants' data contained more than 75% of valid pupillary measures across the whole experiment. Blinks, missing and invalid data points were first linearly interpolated using the interp1 function in Matlab (Matlab 7.11.0). The data was then detrended (to remove slow irrelevant drifts) with simple linear regression within each block. Pupillary responses for each of the conditions of interest (cue-positive; cue-negative; picture-positive; picture-negative) were calculated by subtracting the baseline pupil diameter (first 165 ms, 50 timepoints) from pupil diameter during the trial at each of the consecutive measurement points during the epoch. This time-span of the baseline period is in accordance with prior studies using a similar design (e.g., Silk et al., 2012). These differences were then averaged across trials and across subjects, excluding trials for which 50% or more of the pupil dilation data were missing. In all conditions all trials were retained in at least 96% of the participants. This resulted in four waveforms, each 6 s long (baseline 165 + 5835 ms), that represented averages over the length of the cue and picture periods, separated by condition.

### Data analytic plan

Pupil analyses were conducted in Matlab 7.11.0 by contrasting mean waveforms for cue and picture trials (both positive and negative) at each timepoint along the waveform. Results report mean pupillary response in significant windows.

#### Mood

Paired *t*-tests for the values before (Tpre) vs. after (Tpost) the appraisal paradigm were performed separately for the different subscales.

#### Permutation tests

Significant windows of the pupillary response were detected using permutation, paired-sample *t*-tests (e.g., Blair and Karniski, 1993; Maris and Oostenveld, 2007). Family-wise type I error was controlled for multiple comparisons using the cluster thresholding method discussed in Maris and Oostenveld (2007), using 10,000 permutation samples. A selection criterion (the critical *t*-value with degrees of freedom equal to 54 and α at 0.05, two-tailed) was predefined to select timepoints in each permutated sample, as well as in the original sample. The *t*-values of neighboring selected timepoints were then added to give *t-sum* (Blair and Karniski, 1993), which represented the *t*-value of this empirically generated cluster. While there could be more than one cluster in each permutated sample, only the cluster with the largest *t-sum* (smallest if *t-sum* was negative) was recorded (Maris and Oostenveld, 2007). The *t-sum*s of the 10,000 clusters were then used to define the upper and lower critical values so that the cluster level α was maintained at 0.05, two-tailed. The lower tail was equal to the 2.5th percentile of the negative *t-sum*s, and the upper tail the 97.5th percentile of the positive *t-sum*s, respectively. Finally, the *t-sum*s of the clusters in the original sample were compared against these critical values. Clusters with *t-sums* not bounded by the critical values were declared to show statistically significant differences between the waveforms of interest.

#### Pearson correlations

To explore the relationship between the pupil size changes during the cue and the picture presentation, mean pupillary changes during the significant windows for cue and picture period were obtained and subject to Pearson correlation computations.

#### Partial least squares correlation (PLSC) analyses

To examine if the significant pupillary response differences identified in the cluster-thresholded permutation *t*-test were associated with participants' emotional regulation (ER) strategy, participants' average pupil size and habitual use of cognitive reappraisal and expressive suppression were subject to the Partial Least Squares Correlation (PLSC) analyses (McIntosh et al., 2004; Alin et al., 2009; Krishnan et al., 2011). This method has been used on neuroimaging data to look for physiological and behavioral latent variables (LV) that capture most covariance from the data matrix representing the cognitive conditions, behavioral measures and the physiological measures. Unlike methods using averaged physiological data within predefined time windows, PLSC preserves the spatial-temporal aspects of the physiological data, providing additional information regarding when and where reliable effects are detected. Details of the PLSC are beyond the scope of this report (for further details, see McIntosh and Lobaugh, 2004; Krishnan et al., 2011), but it is essential to note that PLSC decomposes the pupil-by-ER data matrix, R, into two informative Salience vectors containing the LVs that depict the behavioral and the pupillary responses profiles that best characterize R, respectively. The statistical significance of each LV is evaluated using permutation tests with 500 surrogate samples on the singular values of each LV. The stability of each LV is then evaluated using bootstrapping with 1000 bootstrap samples. Whereas the permutation test evaluates if an LV is likely to be due to noise but does not implicate the reliability of the effect, the bootstrapping evaluates at which time (and spatial location) the salience is consistent, i.e., less affected by changes in the underlying sample (McIntosh and Lobaugh, 2004). The absolute value of the bootstrap ratio, which is the salience divided by its bootstrapped standard error, can be understood as a z-score (McIntosh and Lobaugh, 2004; Krishnan et al., 2011).

In the current report, one PLSC was conducted for the cue condition (e.g., epoch following the cue) and another was conducted for the picture condition (e.g., epoch following the target) (both positive and negative), using the PLSC toolbox (http://www.rotman-baycrest.on.ca/). Within each significant LV, the overall patterns between the pupillary changes and the two ER measures were examined by correlating each rating set to the *pupil scores* of the participants. The temporal profile of the pupil scores was then evaluated from the salience vector. As a result, cases like “double negative” could happen: a negative correlation between pupil scores and the ER rating in combination with a negative salience at specific timepoints would imply a positive pupil-behavior correlation at those timepoints. Significance of a LV was determined at *p* = 0.05.

Finally, to address the question if the habitual use of reappraisal and/or suppression is related to the differences in pupillary responses between the cue and picture presentation, a behavioral PLSC was run on the difference waves between the pupillary responses during the two phases (i.e., subtracting the pupillary response during cue from those during picture, separately for each emotion, resulting in two difference waves, from 165 to 6000 ms), with the two ER scores.

## Results

### Self-report data

The mean reappraisal score was 17.14 (*SD* = 4.72) and the mean suppression score was 17.49 (*SD* = 3.62). The internal consistency of both subscales was average: Cronbach's alpha for cognitive reappraisal items is 0.71 and for expressive suppression items 0.73.

### Mood

For the exact scores on the different VAS, we refer to Table 1. Paired *t*-tests revealed differences in mood before vs. after the appraisal paradigm on vigor, *t*_(57)_ = 3.96, *p* < 0.001, depression, *t*_(57)_ = 3.39, *p* < 0.01, and cheerfulness, *t*_(57)_ = 2.32, *p* = 0.02. Participants reported to be feeling less vigorous, less depressed and less cheerful after the appraisal paradigm. They reported no differences in feelings of fatigue, anger, and tension (*t*s < 1.56, *p*s > 0.13).

**Table 1:** **VAS measures (cm) before (Tpre) and immediately (Tpost) the appraisal paradigm**.

	**Tpre *M* (*SD*)**	**Tpost *M* (*SD*)**
Tired	4.71 (2.22)	4.71 (2.29)
Vigor^*^	4.56 (2.05)	4.04 (2.14)
Anger	0.93 (1.36)	1.15 (1.47)
Tension	3.64 (2.36)	3.76 (2.48)
Depression^*^	2.28 (2.37)	1.83 (2.16)
Cheerful^*^	5.09 (1.92)	4.74 (1.93)

* *ps < 0.05*.

### Pupillary response differences

**Figure 1:**
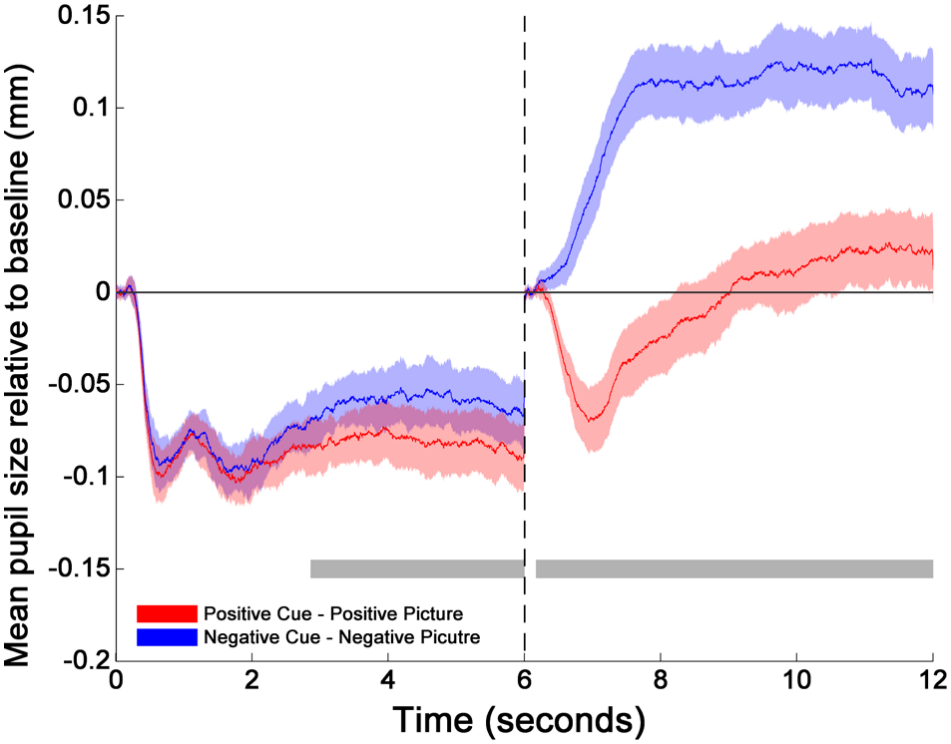
**Changes in pupil size relative to baselines across time for cues and pictures**. The transition of the two phases is marked by the dotted line at 6 s. The two horizontal gray bars mark the time windows within which the differences between positive (red solid line) and negative (blue solid line) emotion trials are statistically significant as indicated by the cluster thresholding method. The colored shades surrounding each mean pupil responses (solid lines) indicate the 95% confidence intervals of the difference between Positive and Negative emotion trials. They are constructed based on the critical paired-sample *t* scores at each time point (Standard Error × *t* scores at the 2.5 and 97.5th percentiles of the permuted *t* distribution). 0–165 and 6000–6165 ms were the baseline periods.

The cluster-thresholded, permutation *t*-tests indicated that the proportional changes in pupil diameter (from baseline) were specifically smaller during the cue-positive condition compared to the cue-negative condition between 2860 and 6000 ms. Moreover, the permutation *t*-tests indicated that pupil diameter during the picture-positive condition was proportionally smaller compared to the picture-negative condition throughout the whole picture epoch (between 165 and 6000 ms; see Figure 1)^2^.

### Pearson correlation between mean pupillary changes during cue and picture

**Figure 2:**
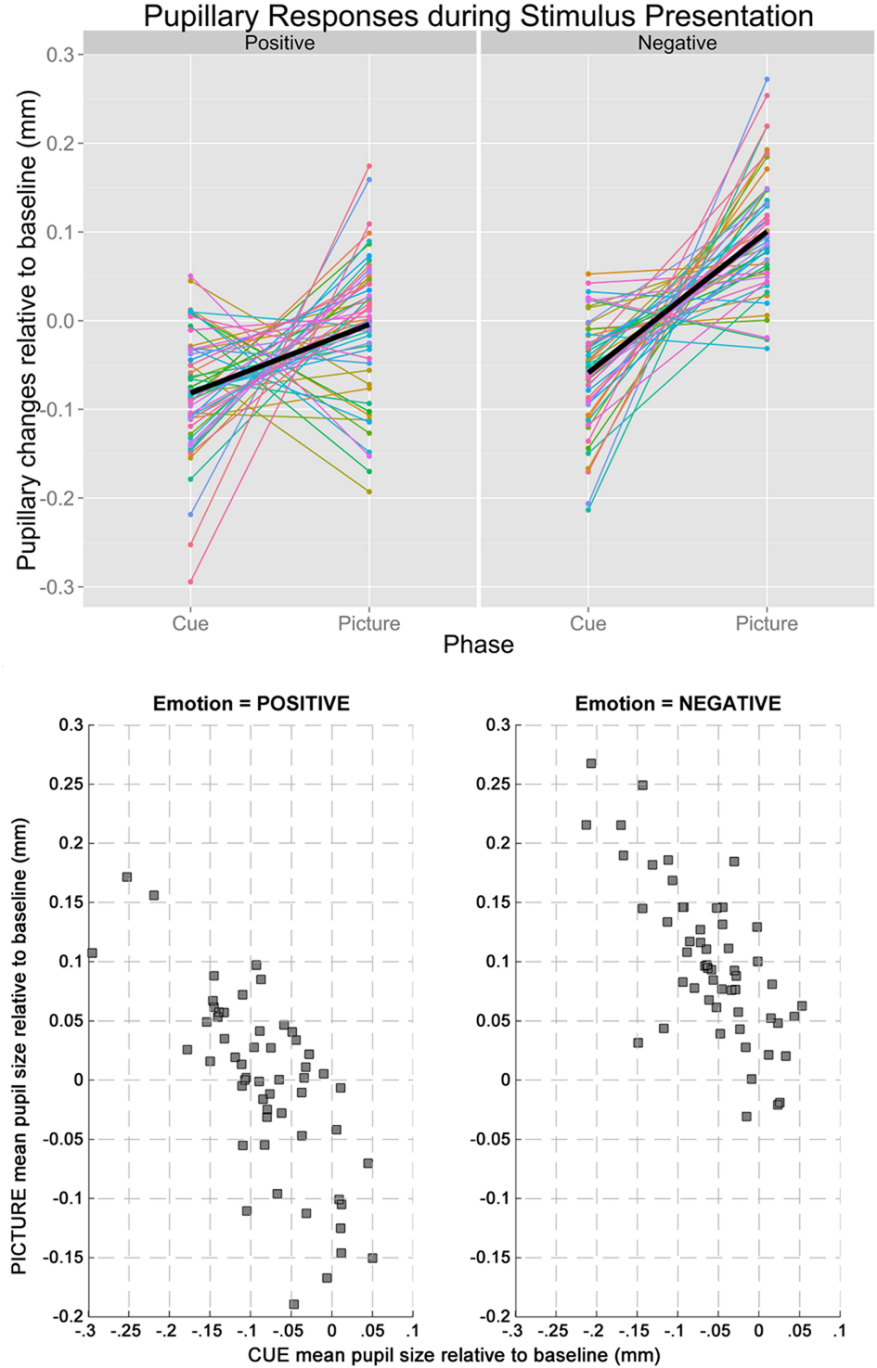
**The upper panel shows the line plot illustrating the changes in baseline-adjusted pupil diameters from cue to picture of individual participants**. Regardless of emotion (left: positive, right: negative), pupillary changes tended to have opposite signs during the two stages of stimulus presentation, explaining the inverse correlations. The lower panel shows the scatterplots between mean pupil sizes during cue and picture when the Emotion of the trials was positive (left) and negative (right).

Based on the results from the permutation *t*-tests, mean pupillary changes from baseline were calculated between 2860 and 6000 ms for cue condition, and between 165 and 6000 ms for picture condition, respectively. The mean pupil diameter was smaller in the cue-positive condition compared to the picture-positive condition, *t*_(54)_ = 4.20, *p* = 0.0001, and smaller in the cue-negative condition compared to the picture-negative condition, *t*_(54)_ = 10.00, *p* < 0.0001. Most importantly, changes in pupil diameter (relative to baseline) during the cue-positive condition were inversely correlated to the changes in pupil diameter (relative to baseline) during the picture-positive condition, *r*_(53)_ = −0.726, *p* < 0.001. The same inverse correlation was observed between cue-negative condition and picture-negative condition, *r*_(53)_ = −0.725, *p* < 0.001. Line plots of individual subjects and a scatterplot of the correlations are plotted in Figure 2, upper and lower panel respectibly.

### PLSC during the cue

**Figure 3:**
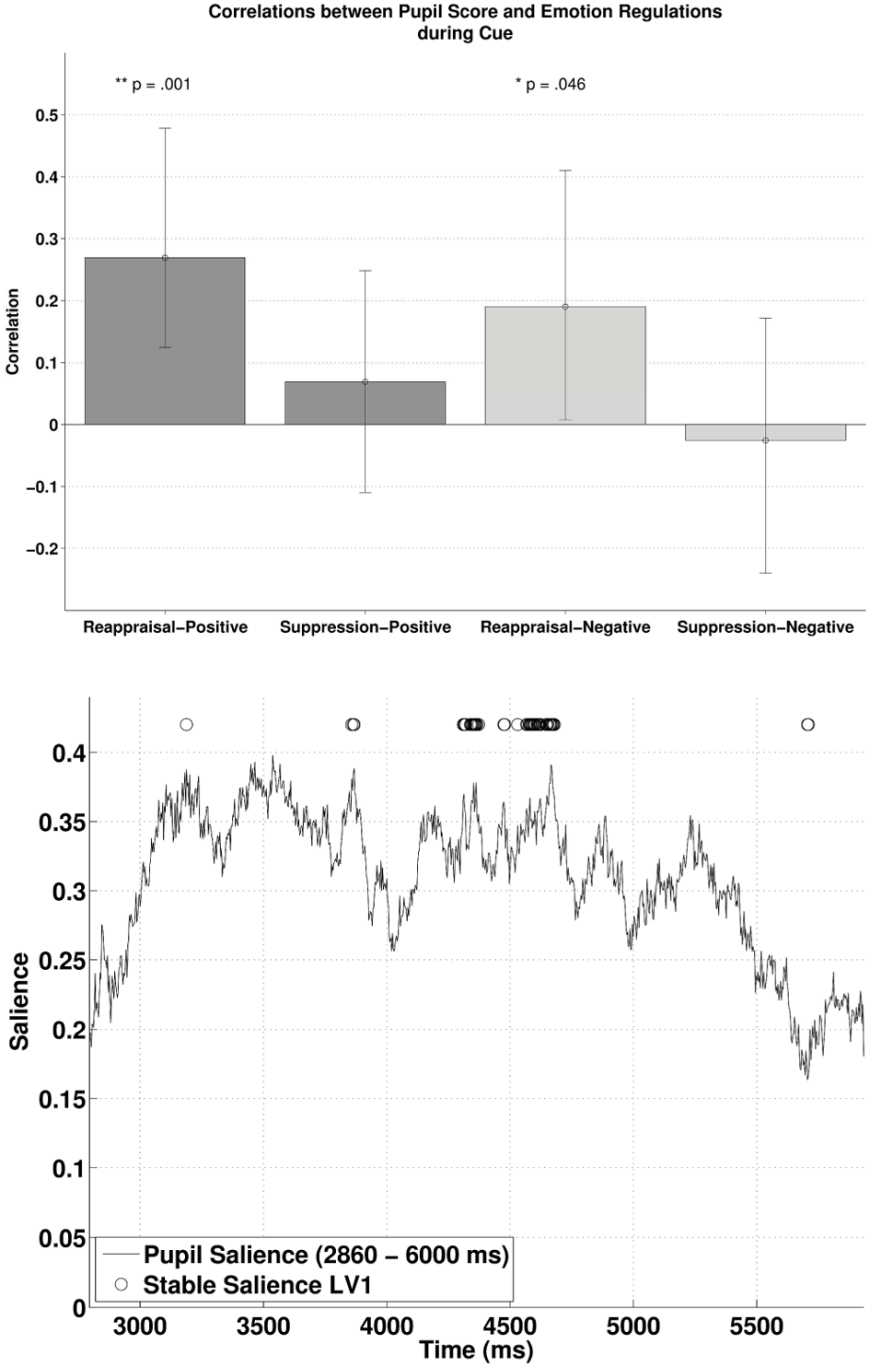
**The upper panel shows the correlations between participants' pupillary responses and the ERQ scores in Latent Variable 1 during cue presentation**. Error bars represent boot-strapped 95% confidence intervals. The lower panel shows that the saliences are in general positive, implying that the reappraisal correlations are interpreted as: the higher the rating, the larger the pupillary responses (or alternatively, a tendency from constriction to dilation).

Subjecting pupillary responses during the cue-positive and cue-negative condition (2860–6000 ms, time window defined by the permutation *t*-tests) and the two ER scores (reappraisal and suppression) into behavioral PLSC yielded four LVs [two Emotions (positive, negative) × two ER scores (reappraisal, suppression)]. Only LV1 attained significance, *p* = 0.014, explaining 96% of the covariance between pupillary changes and the Emotion—ER specification (no trends for other LVs, *p*s > 0.71). We refer to Figure 3 (lower panel) for the salience and stability of LV1 across time. The salience of LV1 was primarily positive. Reliable salience that contributed most to the significant LV (circle markers in Figure 3, with bootstrap ratio >2.40, threshold obtained based on the empirical distribution of all bootstrap ratios) can be observed between 4310–4373 and 4531–4679 ms after cue onset. The relationship between ER and pupillary response captured by LV1 is illustrated in the upper panel of Figure 3. There was a positive correlation between reappraisal scores and pupil size when the cue predicted a positive picture (cue-positive condition), *r* = 0.27, *p* < 0.001, (95% CI = [0.12, 0.48]), and when the cue predicted a negative picture (cue-negative condition), *r* = 0.19, *p* = 0.046, (95% CI = [0.0073, 0.41]). The two correlations were compared with bootstrapped fisher's *z*-test. Sample *z* = 0.43, with 95% CI = [−1.20, 1.86], which included zero, suggesting that the two correlations were not statistically different. This positive correlation between pupillary responses and reappraisal, considered in conjunction with the stable positive salience, implicates that higher habitual reappraisal scores were associated with larger pupillary response when participants were cued for a positive and negative picture (the reverse interpretation is valid as well). For suppression, none of the two correlations were significant, as their 95% CI contained zero.

### PLSC during the picture

**Figure 4:**
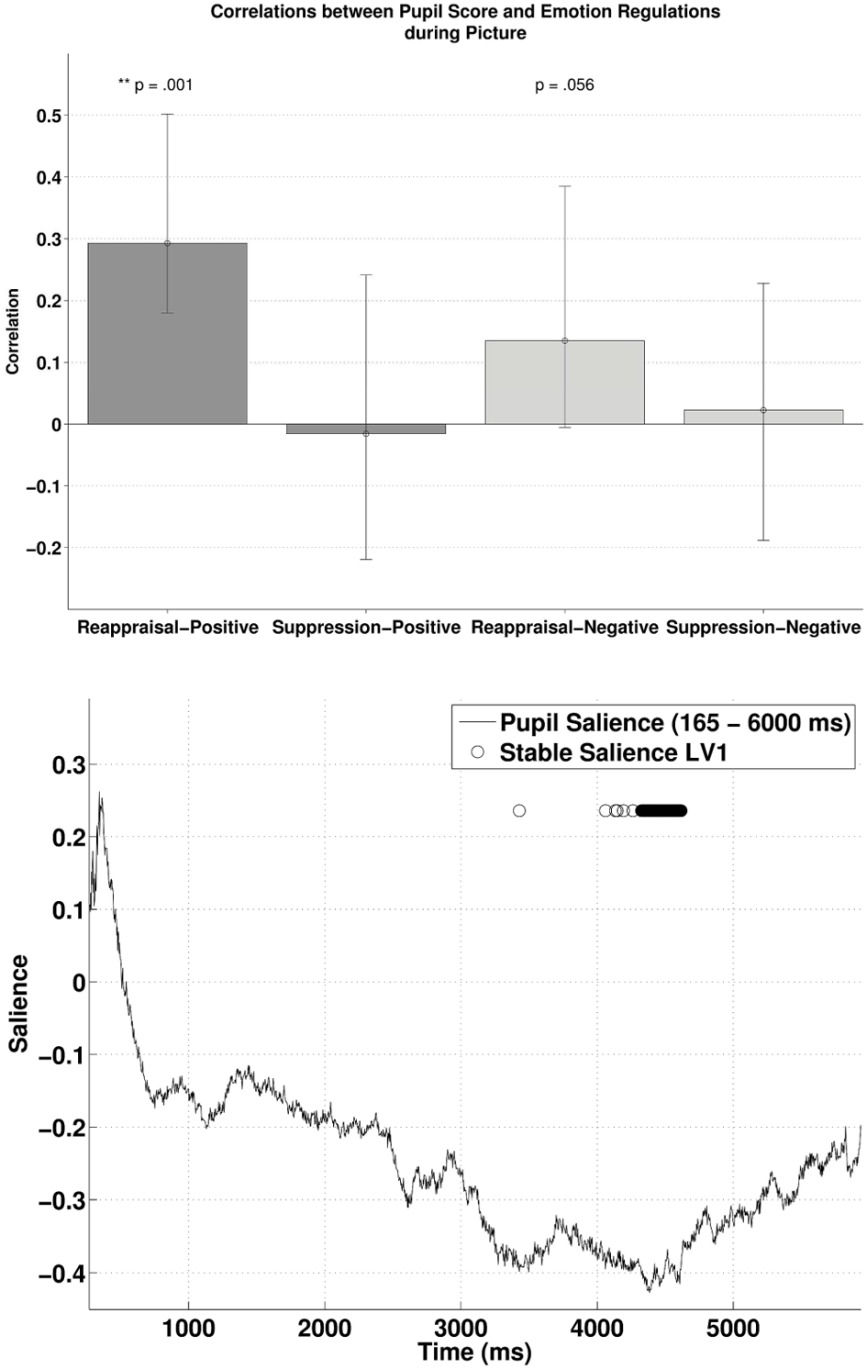
**The upper panel shows the correlations between participants' pupillary responses and the ERQ scores in Latent Variable 1 during picture presentation**. Error bars represent boot-strapped 95% confidence intervals. The lower panel shows that the saliences are in general negative, implying that the reappraisal correlations are interpreted as: the higher the rating, the smaller the pupillary responses (or alternatively, a tendency from dilation to constriction).

Similarly, subjecting pupillary responses during the picture-positive condition and picture-negative condition (165–6000 ms) and the two habitual ER scores into behavioral PLSC yielded only LV1 (out of four) that attained significance, *p* = 0.026, explaining 89.5% of the covariance (no trends for other LVs, *p*s > 0.72). Figure 4 (lower panel) illustrates the time samples with stable salience that contributed most to the significant LV (marked with circles on top of the salience time course with bootstrap ratio < −2.57, threshold obtained from the empirical bootstrap distribution). The salience was primarily negative. The most reliable salience was clustered at 4326–4617 ms. The relationship between pupillary response and the ER measures captured by LV1 is illustrated in the upper panel of Figure 4. There was a positive correlation between reappraisal scores and pupil size when it was a picture-positive condition, *r* = 0.29, *p* < 0.001, (*r* = 0.29, 95% CI = [0.18, 0.50]). This correlation was also positive but only marginally significant during picture-negative condition, *r* = 0.13, *p* = 0.056, 95% CI = [−0.0055, 0.38]). The two correlations were compared with bootstrapped fisher's *z*-test. Sample *z* = 0.84, with 95% CI = [−0.85, 2.087], which included zero, suggesting that the two correlations were not statistically different. This positive correlation between pupillary responses and reappraisal, considered in conjunction with the stable negative salience, implicates that higher reappraisal scores were associated with smaller pupillary change when participants were attending to picture-positive condition and picture negative condition (although the latter was only marginally significant). We observed no reliable correlations between pupil size and suppression. Finally, the opposite signs in salience between the two PLSC analyses also suggest that pupillary changes during the picture were in the opposite direction as during the cue.

### PLSC between cue and picture phase

**Figure 5:**
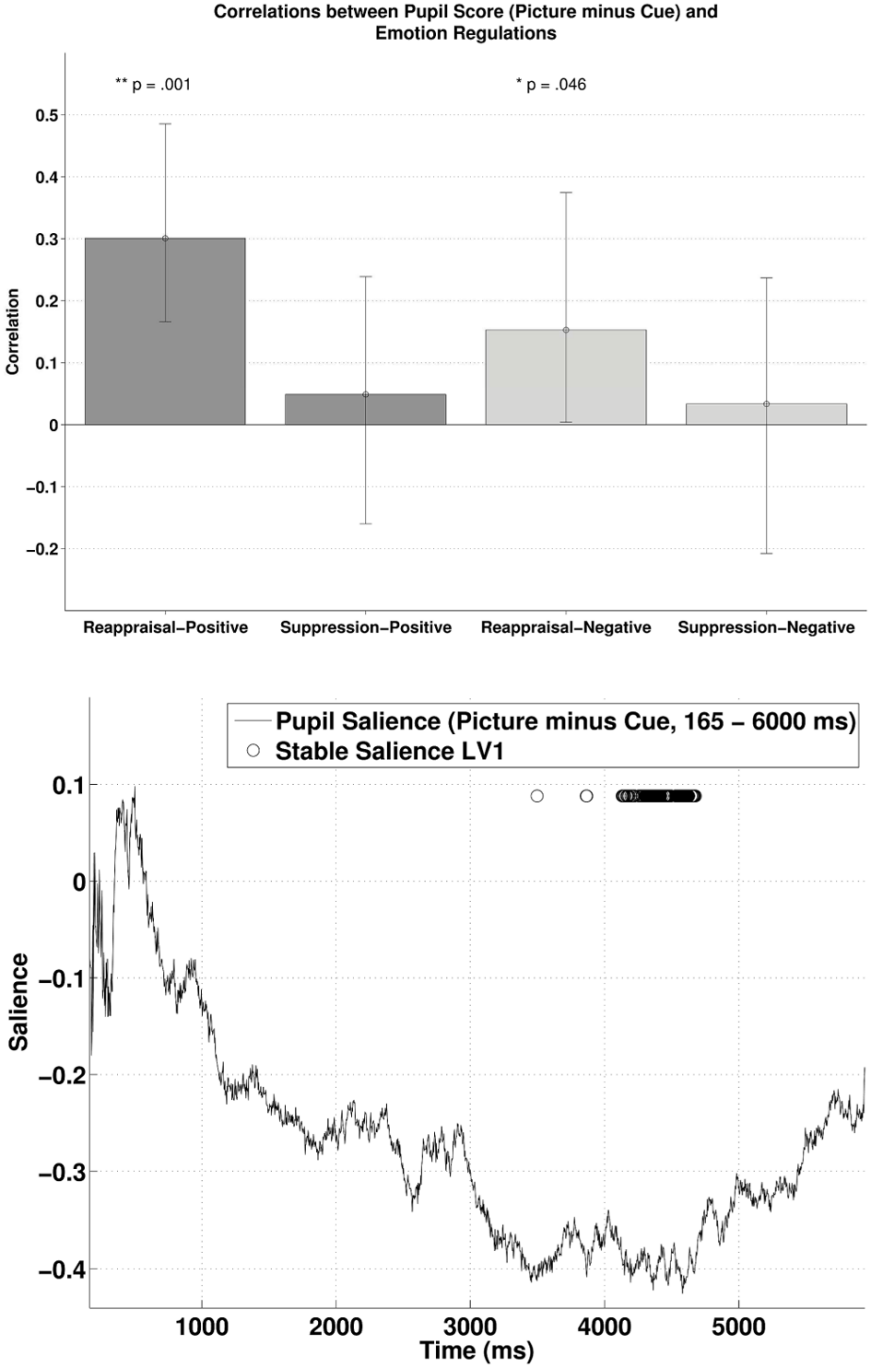
**The upper panel shows the correlations between participants' pupillary responses and the ERQ scores in Latent Variable 1, with the scores derived from the difference in pupillary responses between cue and picture presentation phases (Picture minus Cue)**. Error bars represent boot-strapped 95% confidence intervals. The lower panel shows that the saliences are in general negative, implying that the reappraisal correlations are interpreted as: the higher the scores, the less positive/ more negative the difference in pupillary responses.

The behavioral PLSC on the difference waves (i.e., subtracting the pupillary response during cue-condition from the response during picture-condition) with the two ER scores yielded a significant LV1, *p* = 0.016, explaining 86.34% of the covariance. Figure 5 (lower panel) illustrates the time samples with stable salience marked with circles (bootstrap ratio < −2.57). The most reliable salience was clustered at 4132–4676 ms. As shown in the upper panel of Figure 5, there was a positive correlation between reappraisal scores and pupil size when it was a picture-positive condition, *r* = 0.30, *p* < 0.001, 95% CI = [0.18, 0.50]), or picture-negative condition, *r* = 0.15, *p* = 0.035, 95% CI = [0.0032, 0.38]). The two correlations were again compared with bootstrapped fisher's *z*-test. Sample *z* = 0.79, with 95% CI = [−0.39, 1.83], which included zero, suggesting that the two correlations were not statistically different. The opposite signs in the salience and the correlations demonstrates that higher habitual reappraisal scores were associated with a less positive/more negative pupil size difference between the picture and cue condition. The negative relationship implies that for people who scored highest on habitual reappraisal, the pupillary response during cue would be more positive than during picture, an indication of more effortful processing in the cue as compared to the picture condition. Finally, in line with the analyses above, we observed no reliable correlations between pupil size and suppression.

## Discussion

In this study, we first investigated whether the anticipation of an emotion-eliciting stimulus influences the subsequent online processing of that stimulus. Second, we explored whether the habitual use of emotion regulation strategies (cognitive reappraisal and expressive suppression) was associated with this potential temporal interplay between anticipation and online cognitive processing of emotion-eliciting events. We measured pupillary responses, during the cue and the picture presentation, as a reliable measure of (automatic) arousal associated with effortful processing of emotional material. Overall, we observed that participants were feeling less vigorous, less depressed and less cheerful after the paradigm (no changes for fatigue, anger, and tension), suggesting that the presentation of the emotional images elicited emotional responses.

First, we observed that the pupillary response during the anticipation of an emotion-eliciting stimulus was inversely correlated to the pupillary response during the emotional picture presentation. This observation was evident for both positive and negative emotions (both showing high correlation coefficient, *r*s > 0.7). As can be seen on the line plots (see Figure 2), these inverse associations between cue and picture pupillary responses were not all in the same direction. Half of the participants had larger pupillary responses during the cue and subsequent smaller responses during the picture, and the other participants demonstrated the opposite pattern of cue-picture pupillary responses. These findings demonstrate a reliable temporal interplay between pupillary responses during the anticipation and subsequent online processing of emotional events. Given that larger pupil diameter is a proxy of arousal associated with enhanced mental effort, these findings are in line with the idea that enhanced mental effort during the anticipation of an emotion-eliciting event leads to less mental effort during the online processing of that emotional event. Moreover, also the reverse conclusion can be made, namely less mental effort during the anticipation of emotion-eliciting events is associated with more mental effort during the online processing of the emotion-eliciting event. These findings are in line with the DMC theoretical framework (Braver et al., 2007; Braver, 2012), i.e., that cognitive processing effort during the anticipation influences the effort that is required during the actual confrontation with that event. In other words, the anticipation and subsequent processing of emotional events don't act independently from each other, and there seems an important computational trade-off between both.

Second, these above described individual differences in the temporal interplay between anticipation and online processing of emotional events are found to be associated to habitual tendencies to regulate emotions in daily life. Regression analyses demonstrated that high reappraisal scores predicted enhanced pupillary responses during the anticipation and subsequent smaller pupillary responses during the online processing of emotion-eliciting events (the opposite was observed for low reappraisal scores). The habitual use of reappraisal was also related to the inverse correlation between cue and picture condition, anticipation, and online processing respectively. These findings were evident for both positive and negative emotions, even though the association with positive emotions were stronger compared to the association with negative stimuli (see below). Interestingly, the hypothesis that the habitual use of suppression would be associated with pupillary changes in the picture period was not confirmed, neither for positive nor for negative stimuli. These results are not in line with prior work, which has demonstrated that suppression requires more cognitive effort than reappraisal (Richards and Gross, 2000). These latter authors have demonstrated–using three studies that differed in induction procedures, methods and setting- that cognitive reappraisal and expressive suppression have different cognitive consequences, with the highest cognitive costs (i.e., lapses in memory) for suppression. Emotion regulation strategies were measured both in a controlled laboratory condition and in everyday life. However, in these studies, the dependent variable at the level of cognition (lapses in memory) is not related to emotional arousal. Possibly in our study, because there was no instruction to regulate emotions what so ever, habitual suppressors might have used a cognitive disengagement or distraction strategy leading to less emotional arousal. Another possible reason for these null effects in suppression is that pupil dilation might be an objective measure of autonomic arousal associated with effortful reappraisal (see studies of Urry et al., 2006; Johnstone et al., 2007; van Reekum et al., 2007) but not suppression. All together, given that the reason for inconsistencies between this and prior studies remains unclear (see also Vanderhasselt et al., 2013), further research is needed to explore the association between expressive suppression (both *in vivo* as with a questionnaire) and the anticipation/online processing of emotional material.

How can larger pupil diameter—as a proxy of automatic arousal associated with enhanced cognitive processing effort—be interpreted in light of our present findings? The answer to this question could be found in neuronal activation that is associated to pupil diameter and cognitive processing. Changes in pupil diameter have been associated to neuronal activation in the dorsolateral prefrontal cortex (DLPFC) (Siegle et al., 2011), a brain region which is known to be elevated and sustained during attentional set updating based on contextual information (e.g., a cue) (Vanderhasselt et al., 2006, 2007). Moreover, during the online processing of emotion events, elevated activation in the DLPFC has been associated with a more cognitive effort to regulate emotions (Ochsner et al., 2004), and also to the habitual use of cognitive reappraisal (Drabant et al., 2009). Ochsner and Gross (2005) stated in their review that DLPFC activation is not specific to positive or negative emotional stimuli, but rather refers to cognitive control to regulate responses to an emotional stimulus. Neural regions engaged during emotion regulation are, at least partially, overlapping with those that are involved in cognitive control more broadly (Ochsner and Gross, 2005). Possibly, based on these functional roles of activation in the DLPFC (e.g., attentional set, cognitive control, and emotion regulation), larger pupillary responses during the anticipation of an emotional stimulus reflects sustained attentional set activation to prepare for an upcoming emotional context. This enhanced preparation might subsequently be associated with a reduced need to process the emotional stimulus when being confronted with it. This association between anticipation and online processing is strongest for habitual reappraisers. This latter ER strategy is found antecedent-focused, namely that is starts operating before appraisals give rise to full-blown emotional responses (Gross, 2002; Sheppes and Gross, 2011), and the emotion processing gradually diminishes over time when the emotional stimulus is still presented (Goldin et al., 2008). Yet, even though there is an association between reappraisal ability and frequency (McRae et al., 2011), the association between pupillary response and emotion regulation in this study is challenging because participants were not explicitly instructed to regulate their emotions in any way. Participants were asked to respond naturally and were not aware of the real purpose of the study. Possibly, pupillary responses in this study may reflect a human self-regulation, which is most obvious in high reappraisers, referring to an increase in arousal associated cognitive effort when anticipating associated with a decrease of this effort during the subsequent online processing of emotion-eliciting events.

Even though the absolute pupil diameter of negative stimuli was in general larger as compared to the pupil diameter of positive stimuli (as can be expected from prior research, e.g., Bitsios et al., 2004), the interplay between anticipation and online processing of emotional stimuli was similar and strong for both emotions (*r*s > 0.7). It should be emphasized that—although not statistically different- habitual reappraisal scores were more strongly related to the cue/picture interplay for positive compared to negative pictures. Enhanced cognitive effort to embrace positive emotional stimuli, as soon as they are expected, to later on reduce the processing of it, seems therefore typical for high reappraisers. Differences between positive and negative emotions could, however, be due to differences in arousal (see below). Interestingly, our results demonstrate that—overall—the interplay between anticipation and online processing for positive and negative stimuli is reversed for high and low reappraisal scores. This means that high reappraisers, who are more likely to have high emotional well-being, are spending cognitive effort when anticipating than online processing emotional stimuli. Because the frequency with which one uses cognitive reappraisal is an important factor for greater emotional well-being (Gross and John, 2003; McRae et al., 2011), the computational trade-off between anticipation and online processing might be important marker for this well-being. Moreover, given that individuals who tend to use reappraisal in daily life have improved cognitive control ability to reduce emotional effects when confronted with the stimulus (Cohen et al., 2011), adequate anticipation might play an important role in this matter. Further research might therefore want to explore how this anticipation/online processing of emotional events is associated to variations in emotional well-being. However, further research is also needed to investigate the differential effects for positive and negative information in habitual reappraisal. Furthermore, the correlation with the habitual use of reappraisal was strongest after a couple of seconds in both the cue (most positive correlation between 4310–4373 and 4531–4679 ms) and the target (most negative correlation between 4326–4617 ms) period. Also, the salience in both conditions seems to demonstrate a parabolic distribution, with a building up from the beginning and a decrease toward the end. This means that further research should also consider the temporal dynamics of the effect of reappraisal during the anticipation and online processing of emotional information.

It should be stated that positive and negative pictures were not only different on pleasantness, but also on normed arousal ratings (negative being more arousing than positive images). This is reflected in the picture condition by more dilatation for negative compared to positive images. Consequently, the impact of arousal on pupil dilatation is not controlled between positive and negative image categories. This is a limitation of our study, and further work should take this arousal variable into account in order to investigate the interplay between anticipation and online processing of emotional events. Nevertheless, results for both positive and negative valenced images demonstrated a similar pattern of results, and consequently no valence specific conclusions were made. Even though the cue image (depicting the word “positive” or “negative” on a neutral background) is neutral by nature, the fact that a cue for a negative image was always followed by a negative image, and a cue for a positive image was always followed by a positive image, seems to have primed the emotions that were anticipated. This is because the pattern of arousal in the cue condition was similar as in the picture condition (i.e., more dilatation for negative compared to positive information), and validates the emotion specificity of the cue foreseeing the target. The computational trade-off between anticipation and online processing is therefore not due to arousal differences in the cue or target. Yet again, no differential conclusions regarding positive and negative emotions are drawn, only regarding the interaction between anticipation and online processing for both valences. Another limitation of our study is that participants reported to feel less vigorous, depressed and cheerful at the end of the experiment. Even though we observed no changes for fatigue, anger and tension, these former changes in mood could simply be due to the time spent in the lab. If so, these ratings do not allow to verify that participants responded emotionally to the stimuli, and were able to modulate their emotional response according to their habitual emotion regulation strategy. Future research should include additional behavioral or physiological measures of subjective mood and/or arousal.

To conclude, larger pupillary responses during the anticipation of an emotional stimulus might be indicative of a sustained attentional set activation to prepare for an upcoming emotional stimulus, which seems to be related to a reduced need to cognitively process that emotional event. Individual differences in the habitual use of cognitive reappraisal are associated with the temporal interplay between anticipation and online processing of emotional events.

## Author contributions

Marie-Anne Vanderhasselt, Jonathan Remue, and Rudi De Raedt designed the study. Marie-Anne Vanderhasselt and Jonathan Remue acquired the data. Marie-Anne Vanderhasselt and Kwun K. Ng analyzed the data. Marie-Anne Vanderhasselt, Jonathan Remue, Kwun K. Ng, and Rudi De Raedt wrote the article. All authors reviewed the article and approved its publication.

### Conflict of interest statement

The authors declare that the research was conducted in the absence of any commercial or financial relationships that could be construed as a potential conflict of interest.

## Footnotes

^1^The following IAPS pictures for negative emotions were included: 1019, 1205, 1300, 2117, 2800, 2811, 2911, 3170, 3530, 3550, 6021, 6212, 6213, 6250, 6550, 6560, 6570, 8230, 8430, 8480, 9040, 9181, 9250, 9252, 9254, 9300, 9423, 9571, 9594, 9635. The following IAPS pictures for positive emotions were included: 1340, 1441, 1659, 1710, 1920, 2040, 2045, 2151, 2158, 2306, 2314, 2332, 2339, 2340, 2352, 2373, 2392, 2395, 2550, 4542, 4599, 4625, 4641, 5829, 5831, 5833, 5836, 7325, 8350, 8540.

^2^The figures showing pupillary responses during the cue and picture periods, separately for high and low reappraisers and suppressors, can be found in the supplementary material section.

## Supplementary material

To visually explore the reported relations between emotional regulation ratings and pupillary responses in a more visually tractable manner, participants were grouped in to low and high scorers of each ERS (Reappraisal/ Suppression). Groups with low and high scores were defined as participants having their ERS ratings at or below the 25th percentile and at or above 75th percentile of the sample, respectively. This resulted in a sample size of 15–19 per group. Data are plotted separately for each rating and phase, resulting in four figures, i.e., Reappraisal-Cue, Reappraisal-Picture, Suppression-Cue, and Suppression-Picture. In each figure, average responses for each emotion (Blue: positive, Orange: negative) and group (Solid: Low scorers, Dotted: High scorers) are depicted.
