# Supplementary figures and images for: The interplay between the anticipation and subsequent online processing of emotional stimuli as measured by pupillary dilatation: the role of cognitive reappraisal
Source: Front Psychol. 2014 Mar 13;5:207. doi: 10.3389/fpsyg.2014.00207 (PMC3952078; doi:10.3389/fpsyg.2014.00207)

**Pupil Size Time Course during Picture for  
Low (<25% percentile) and High (>75% percentile) Reappraisers**

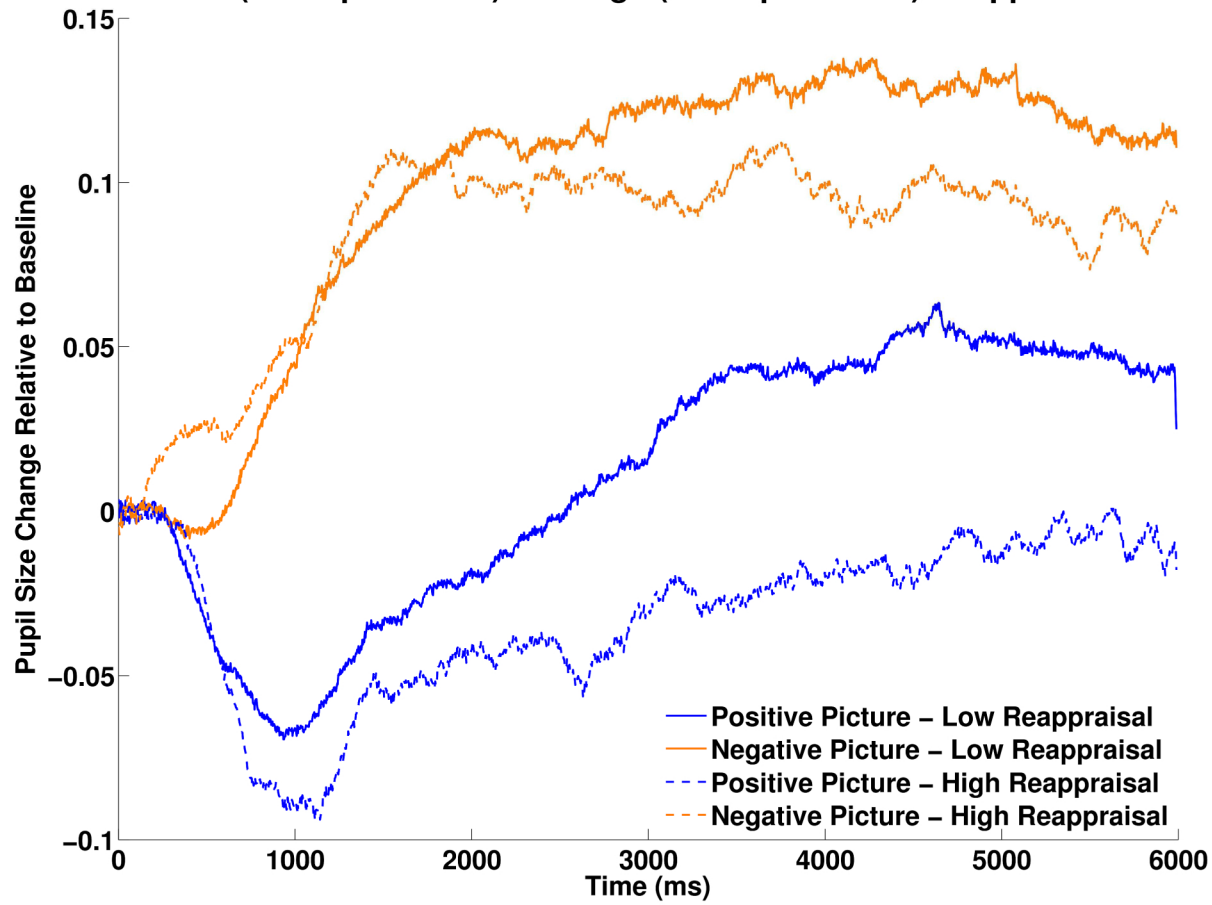

Supplement: Supplementary file 1 [file Presentation1.ZIP › S2.pdf]

# Pupil Size Time Course during Cue for Low (<25% percentile) and High (>75% percentile) Suppressors

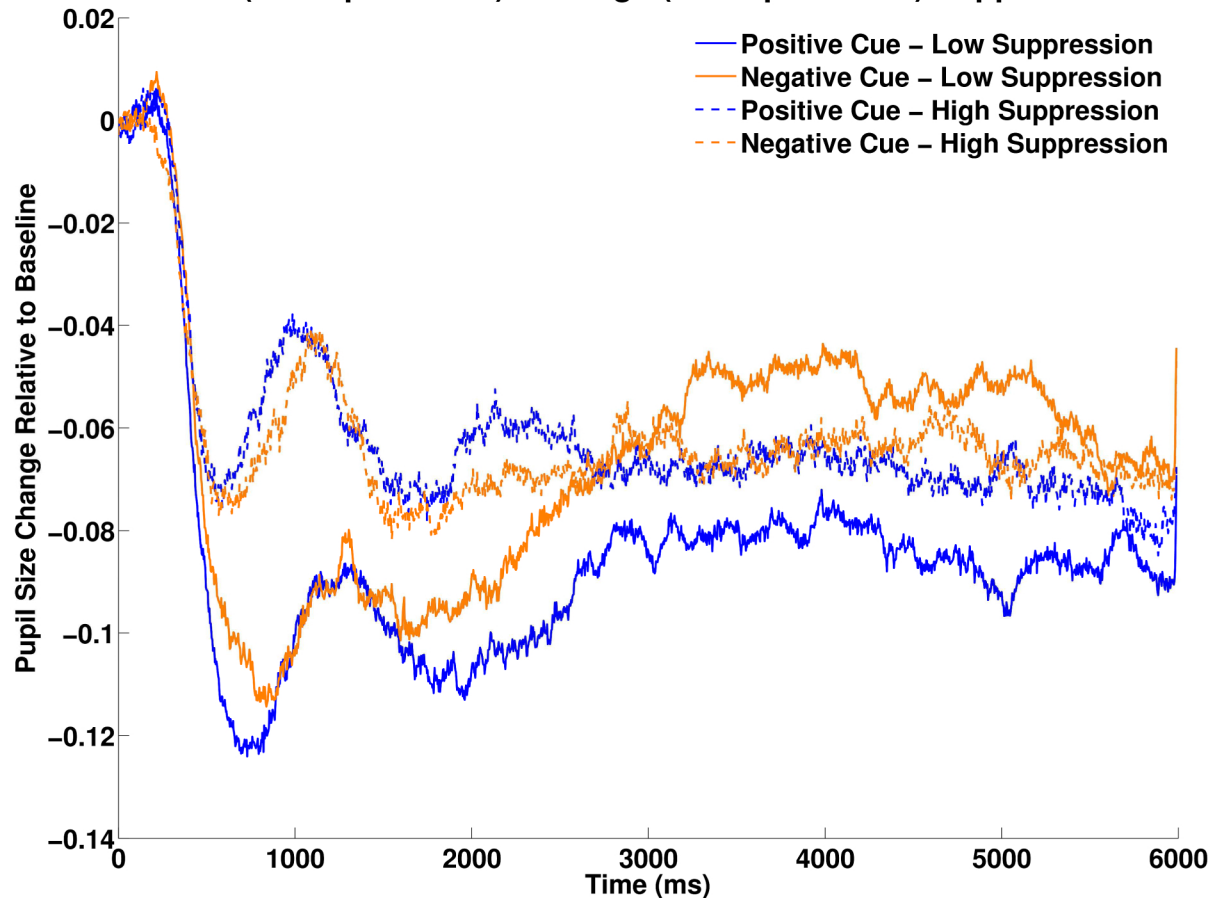

Supplement: Supplementary file 1 [file Presentation1.ZIP › S3.pdf]

**Pupil Size Time Course during Picture for  
Low (<25% percentile) and High (>75% percentile) Suppressors**

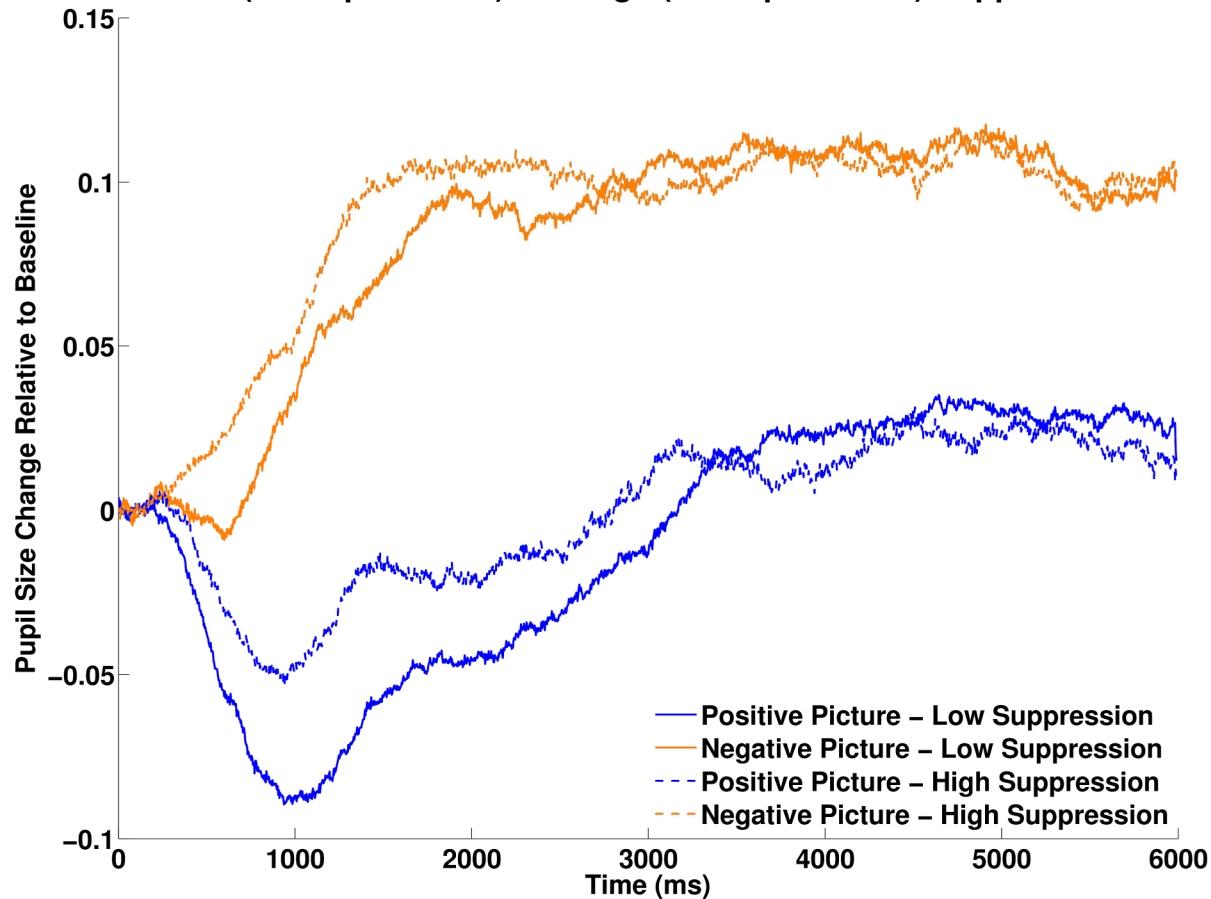

Supplement: Supplementary file 1 [file Presentation1.ZIP › S4.pdf]

# Pupil Size Time Course during Cue for Low (<25% percentile) and High (>75% percentile) Reappraisers

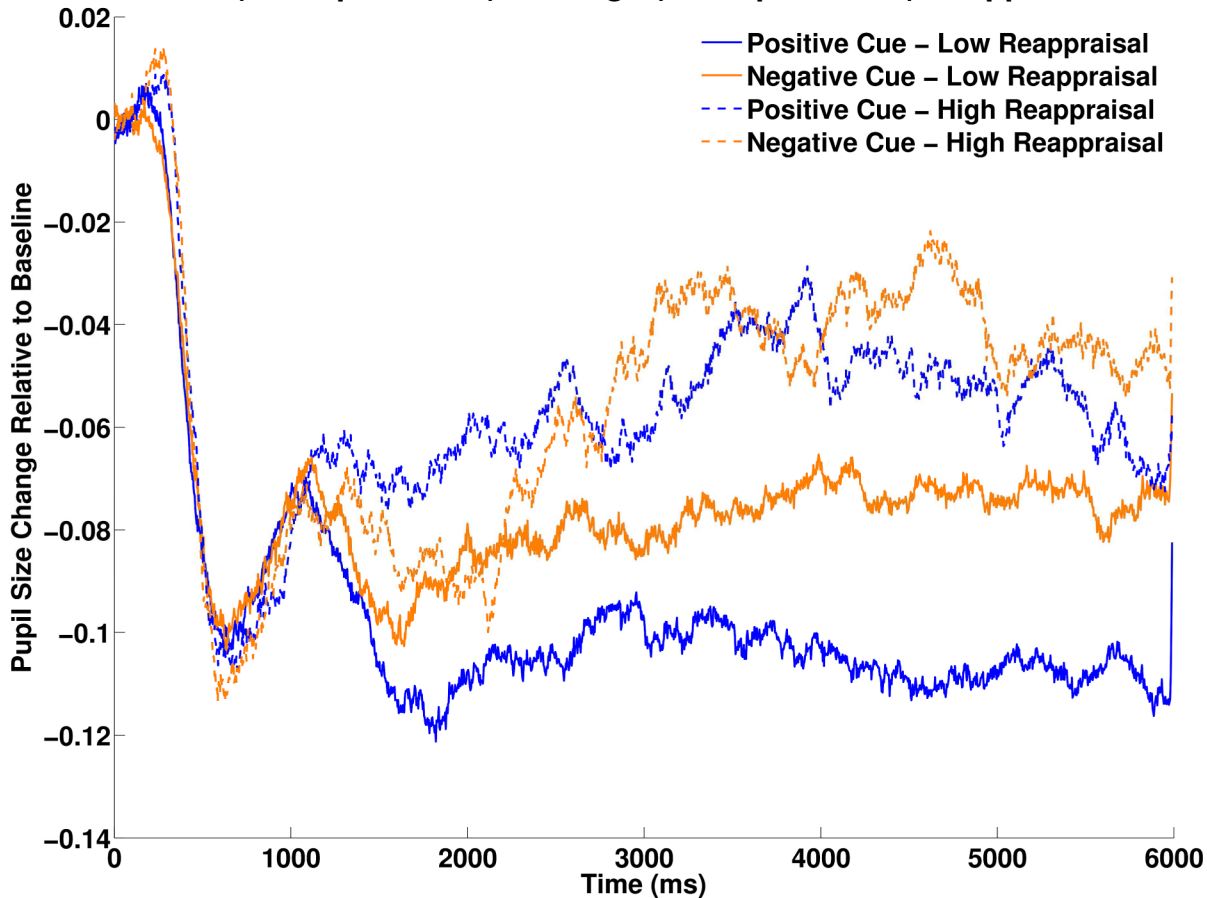

Supplement: Supplementary file 1 [file Presentation1.ZIP › S1.pdf]
